# Implementing contingency management to improve engagement among justice-involved clients referred for substance use disorder services

**DOI:** 10.3389/fpubh.2026.1907828

**Published:** 2026-08-19

**Authors:** Bryan Hartzler, Alanna Feltner, Chris Wig, Jason Tran, Mandi Nugent, Jennifer Verbeck, Kelsey Payne, John W. McIlveen

**Affiliations:** 1 Department of Psychiatry and Behavioral Sciences, Addiction, Drug & Alcohol Institute, University of Washington, Seattle, WA, United States; 2 Emergence Addiction and Behavioral Therapies, Eugene, OR, United States; 3 Health Systems Division, Oregon Health Authority, Salem, OR, United States

**Keywords:** community implementation, contingency management, criminal justice, health services, substance use disorders

## Abstract

**Introduction:**

Criminal justice involvement is prevalent among persons with substance use disorders (SUDs), for whom contingency management (CM) has been shown to increase treatment adherence. To support CM implementation by SUD organizations serving justice-involved clientele, effective technical assistance (TA) approaches are needed. This community case study details the sustainable implementation of CM that was achieved through support of an empirically supported TA approach.

**Methods:**

In the context of an interagency partnership, a university-based team employed a phased TA approach to support CM implementation by an SUD organization. This included the collaborative design of customized CM programming for clients referred by a local treatment court, with reinforcement targeting service initiation and engagement. Treatment staff completed preparatory training and skills-based coaching, and prepared facility systems (e.g., accounting, clinical records, and staff supervision) for a 6-month implementation period. Independent evaluation of clinical impacts compared CM-enrolled clients (*n* = 53) with a historical control group (*n* = 212). An eventual decision about sustainment of the CM programming among routine services was elicited from SUD organization leaders.

**Results:**

All 10 treatment staff exceeded *a priori* benchmarks for CM knowledge and skill at the conclusion of the training and coaching processes. Direct evidence of the CM programming’s clinical utility includes its influence in more than doubling the completion rate for intake assessments and increasing group therapy attendance by 11%. A secondary therapeutic benefit to the milieu was an 11% increase in the CM-enrolled clients’ fulfillment of court-assigned SUD service requirements. This set of effects, along with a positive overall implementation experience, informed organization leaders’ decision to sustain the CM programming within routine services thereafter.

**Discussion:**

This community case study demonstrates how SUD organizations may sustainably implement CM programming customized to local clinical needs and resources. Key aspects of the interagency partnership, funding for implementation costs, and the eventual sustainment decision are presented. We hope this will aid others in implementing CM to address local challenges posed by justice-involved clientele.

## 1 Introduction

Criminal justice involvement is a long-recognized prevalent circumstance among persons with a substance use disorder (SUD), and one that affects the course and quality of treatment services they receive (1). Similarly, substance-related problems are among the salient criminogenic predictors of recidivism commonly serving as focal therapeutic targets for such justice-involved treatment populations (2). This nexus of health and legal issues constitutes a compelling and persistent public health challenge in the United States. In economic terms, the expense of its “War on Drugs” is staggering, with corresponding budget allocations in each of the past 2 years—encompassing both the Biden and Trump administrations—falling reliably within a range of $44.5–46.1 billion (3, 4). To justify continued federal expenditure, there is demand for continued improvement in the quality of care provided, including SUD services received by justice-involved treatment populations. Thus, community-based implementation of empirically supported treatments that address SUDs is sorely needed. So too are effective methods whereby local uptake of such treatments is facilitated through technical assistance (TA) processes, which typically encompass tailored educational and consultative services from a subject matter expert. Due to the range and complexity of implementation support services needed to disseminate empirically supported treatments, it is common for organizations to seek implementation support from intermediary purveyor organizations that bridge gaps between academic knowledge of such SUD treatments and their practical, real-world application (5).

Historically, treatment courts have offered a primary referral avenue for justice-involved clients to SUD treatment. As described by Prendergast and colleagues (6), such courts “*introduced a new model……early identification and referral of drug-involved defendants to community-based treatment following arrest or conviction, a specialized court docket, close integration of judicial supervision and treatment, a non-adversarial approach, frequent monitoring by the judge for compliance with treatment, use of graduated sanctions and rewards, and frequent drug testing” (pg. 126).* In their review of SUD service referral of justice-involved individuals, Friedman and colleagues (7) found that such referrals typically provided linkage of clients with cognitive-behavioral approaches like Moral Reconation Therapy (8), drug testing, and mutual-help groups. Unfortunately, justice-involved individuals were found to be referred to SUD services that infrequently incorporated the structured, non-punitive application of operant conditioning principles that define contingency management [CM; (9)]. Findings from Friedman et al.’s (7) early review spurred several subsequent studies of CM programming with justice-involved populations that demonstrated feasibility (10) and clinical effectiveness (11, 12). These studies provided the basis for subsequent advocacy that CM programming with justice-involved populations be tailored to the unique needs of local settings and the communities they serve (13). This sentiment persists today, with the prospect of optimizing SUD services for justice-involved clients among the most compelling reasons for broad dissemination of CM to SUD service organizations.

Although recent attention to CM often highlights its targeted reinforcement of stimulant abstinence (14), a comprehensive review of its study in SUD settings that now spans more than half a century reveals the application of CM to wide-ranging clinical targets. Similarly, this has also encompassed procedurally diverse CM paradigms, each typically defined by the nature of the reinforcers a client earns (e.g., prizes, vouchers, and setting privileges) by demonstrating targeted treatment-adherent behavior (9). Meta-analyses of CM in SUD treatment settings reliably report medium-sized mean effects (15–17), with CM recognized among established behavior therapies as a salient candidate for increasing treatment adherence among persons with diagnostic SUD profiles that are common among justice-involved populations (18, 19). Admittedly, findings of clinical utility in initial CM trials that involved treatment courts were modest (6, 20), though this has since been attributed to poor design features in the involved CM protocols (11)—such as their overly narrow targeting of substance abstinence at the exclusion of reinforcing other targets that reflect client engagement in supportive SUD services. For some SUD treatment settings and circumstances, the latter may better align with an inclusive treatment philosophy that recognizes the greater diversity in many potentially beneficial recovery pathways for addiction (21).

In initial efforts to disseminate CM programming to the SUD treatment community, a host of well-documented barriers were identified. As reviewed by Hartzler and colleagues (22), these included fiscal concerns (e.g., purchase of material reinforcers and staff training/supervision costs), logistical challenges (e.g., complicated reinforcement schedules and disruption to existing clinical services), and philosophical objections (e.g., nonclinical origin and perceived inefficacy or disadvantage among clinicians). Years later, these barriers were compounded by emerging policy-focused concerns (23), implicating federal anti-kickback statutes and annual incentive thresholds. Fortunately, large-scale implementation studies (24–26) increased understanding of strategies for overcoming these collective challenges, and underscored the value of corresponding TA services. In 2025, the Substance Abuse and Mental Health Services Administration released a CM advisory report (27) that provides its grantees with specific guidance on wide-ranging issues, including recognition that implementation efforts by settings offering SUD services will often benefit from consideration of local clinical needs and resources. This is conceptually congruent with the replicated successes of a comprehensive TA approach that has facilitated sustained implementation of customized CM programming at opioid treatment programs over the past decade (28–31). Application of this TA approach, and consequent documentation of real-world implementation success in SUD settings that treat justice-involved clients will provide models for others to emulate in future implementation efforts. To that end, the current report offers an organization-level community case study of CM implementation by an SUD treatment setting with an established treatment court referral pathway for many of its service recipients.

## 2 Context

Born of an interagency partnership, this community case study is part of a continuing effort to support the implementation of CM programming at community-based SUD treatment organizations in the state of Oregon. Originating in 2020, the partnership—involving the Single State Authority and a university-based team contracted to offer an empirically supported package of CM-focused TA services—has persisted under the auspices of three State Opioid Response (SOR) grant cycles. It continues to address clinical challenges posed by opioid and stimulant use disorders at SUD treatment organizations, via SOR-sponsored delivery of a package of TA services for which the feasibility and sustainable implementation of customized, clinically useful CM programming has been established, refined, and replicated (28, 29, 31). This community case study recounts the benefits of this interagency partnership to a particular SUD treatment organization. Each of the three involved entities is now described in greater detail.

### 2.1 Oregon health authority (OHA)

Overseen by a nine-member Oregon Health Policy Board, OHA, in its capacity as the single-state authority, works toward statewide health reform to contain costs while improving access to and quality of care for the citizens of the state of Oregon. For the CM implementation effort described in this report, OHA served as the sponsor—and in this capacity, it specifically provided fiscal support to the local SUD treatment organization by funding its receipt of the noted package of CM-focused TA services over the course of a calendar year. Notably, this did not include the purchase of the tangible reinforcers that would be distributed to clients upon demonstration of targeted treatment-adherent behaviors. Instead, all implementation costs for such tangible reinforcers were supported entirely by the SUD treatment organization, enabling a more authentic and autonomous implementation experience.

### 2.2 Emergence addiction and behavioral therapies (emergence)

Based in the city of Eugene, this non-profit treatment organization serves Oregon’s Lane, Linn, and Benton counties. A trusted resource for community members seeking treatment and recovery for substance use, gambling, and mental health disorders, Emergence services are grounded in principles of evidence-based practice, cognitive-behavioral therapy, and holistic care. During this implementation effort, its SUD service facility in downtown Eugene employed an interdisciplinary staff of 10 individuals, consisting of two clinical supervisors, six counselors, a treatment court liaison, and an administrative specialist—all serving a daily census of approximately100 individuals with heterogeneous SUD diagnoses. It should be noted that the focal intensive outpatient, peer support, and recovery-focused SUD services provided at this facility principally serve justice-involved clients upon their referral from treatment courts in the noted Oregon counties; however, these SUD services are also available to individuals who self-refer from the community. Historically, the facility had struggled to engage justice-involved individuals in its SUD services, with poor rates observed in both initial intake completion and subsequent group therapy attendance among treatment court referrals. This persisting circumstance spurred the organizational goal of Emergence leaders to seek implementation support for CM programming that would be responsive to the local clinical needs posed by treatment court referrals. By virtue of its team of experienced clinicians and its organizational commitment to compassionate care, Emergence supports its neighboring Oregonians as they take their initial steps in recovery.

### 2.3 Center for advancing addiction health services (CAAHS)

Based at the University of Washington, CAAHS maintains a portfolio of funded projects unified by a mission to accelerate implementation of useful treatment practices for persons with SUD. Typically, this involves the provision of TA, broadly defined as tailored educational and consultative services, to treatment organizations and the workforce they employ. Such TA services are conceptualized according to a three-tiered rubric promoted by the Substance Abuse and Mental Health Services Administration [SAMHSA; (32)] consisting of: (1) universal TA, defined as resource-sharing or one-time events that offer orienting information to promote workforce awareness of a useful treatment practice like CM; (2) targeted TA, defined as learning processes to increase workforce members’ knowledge, skill, and readiness to implement a useful treatment practice like CM; and (3) intensive TA, defined as longitudinal and systems-level support to organizations to coordinate implementation of a useful treatment practice like CM by the workforce members they employ. Within this three-tier rubric, the efforts described herein constitute intensive TA.

## 3 Detail

### 3.1 CM-focused TA approach

The delivery of this comprehensive, empirically supported CM-focused TA approach is rooted in the widely cited Exploration-Preparation-Implementation-Sustainment (EPIS) framework (33). It collectively occurs over the course of a 12-month period, which for the noted interagency partnership involving Emergence corresponded to the 2023 calendar year. This involved a chronology of the four EPIS phases outlined in Table 1, each of which is further described in this subsection of the community case study.

**Table 1:** Chronology of activities in CM-focused technical assistance approach.

Phase	Implementation activities
Exploration Months 1–2	*Collaborative design process* Initial meeting of representatives from the single state authority (OHA), leadership of the community treatment organization (Emergence), and technical assistance provider (CAAHS) to discuss implementation goals and timeline Completion of online “decision-maker” training module by Emergence leaders with initial drafting of parameters for customized CM programming Iterative process of CM programming review and revision among Emergence and CAAHS representatives, eventuating in its consensus finalization
Preparation Months 3–5	*Training and coaching of clinical staff, preparation of setting systems* Identification of SUD facility staff to serve as a local implementation team, with subsequent meetings to discuss and prepare its systems for logistics of implementing CM programming Completion of online “clinical supervisor,” “direct-care staff,” and “administrative support staff” training modules by relevant SUD facility staff Participation of staff in a virtual coaching-to-criterion process eventuating in individual assessments with live trainer scoring and feedback until CM fidelity criterion is reached Availing of electronic library of tailored CM resources as reference material for SUD facility staff during their future implementation efforts
Implementation Months 6–11	*Provisional implementation of CM programming* Emergence leadership identification of start date of pilot implementation period, and initiation of this period of active implementation of its customized CM programming Recurrent consultative meetings of facility leaders and CAAHS representative offering trouble-shooting for any challenges in ongoing implementation efforts
Sustainment Month 12	*Examination of local clinical effectiveness, elicitation of sustainment decision* Examination and discussion of the facility-specific implementation costs, staff experiences, and local clinical effectiveness of CM programming by Emergence leadership and CAAHS Writing and distribution of corresponding summary report by CAAHS for OHA and Emergence leadership, highlighting both process description and data-based findings Final meeting involving independent facilitation of Emergence leadership decision about the prospect of sustaining, adapting, or discontinuing CM programming

Activities of this CM-focused TA approach are presented as phased delivery per the Exploration–Preparation–Implementation–Sustainment (EPIS; Aarons et al. al, 2011) implementation framework during the span of a calendar year. Online training modules are freely available through the SAMHSA HealthEKnowledge Repository.

#### 3.1.1 Exploration phase

In the initial 2 months of this full-year effort, the collaborative design of CM programming (28) was undertaken—a process that rests on principles of user-centered design (34). These principles include a focused understanding of the needs of a given product’s intended end-users and the context in which they will use the product, continuous involvement and solicitation of end-user feedback throughout the design of the product, and particular attention to end-users’ experience of product usability and accessibility. At a theoretical level, Chambers and colleagues’ (35) dynamic sustainability framework stipulates that the continual application of such user-centered design principles will promote sustainable therapy implementation—and multiple published examples link this to successful CM sustainment (28, 31, 36). In this process, the UW CAAHS subject matter expertise and the local clinical expertise of Emergence leadership were pooled via a series of meeting discussions to reach consensus on the core CM programming parameters (i.e., eligible clients, target behavior, earned reinforcers, and reinforcement paradigm). These meeting discussions encompassed elicitation of Emergence clinical needs and resources, review of empirically supported CM paradigms, drafting and revision of CM programming examples, and calculation of prospective maximal per-client earnings and other implementation costs. While CAAHS outlined a set of viable options and offered recommendations for specific features to maximize clinical impact, the eventual core parameters of the customized CM programming to be implemented were ultimately chosen by Emergence leadership.

#### 3.1.2 Preparation phase

At the outset of this 3-month phase, a local implementation team comprised of Emergence staff was assembled to prepare facility systems (i.e., accounting, clinical documentation, staff supervision, and program evaluation) for implementation procedures. Meanwhile, role-specific online training modules were completed by “decision-makers,” clinical supervisors, direct-care staff, and administrative support staff to gain a conceptual foundation in CM principles and practices. At the conclusion, each online training module presented the respondent with a 10-item multiple-choice knowledge test to demonstrate the conceptual readiness of individual Emergence staff members.

As further preparation for CM implementation, staff participated in a 6-h virtual coaching-to-criterion process (37) facilitated by the lead author, a clinical psychologist and established subject matter expert. The process consisted of three 2-h group sessions focused on the delivery of CM programming, with emphasis on observational and experiential learning via trainer demonstration and behavioral rehearsal via role-play and other forms of simulation. Through prior completion of online training modules, staff became oriented to the six CM Competence Scale [CMCS; (38, 39)] fidelity domains: (1) notification of earned reinforcers, (2) planning for prospective reinforcers, (3) delivery of earned reinforcers, (4) assessment of client interest in reinforcers, (5) communication of social reinforcement, and (6) linkage of the target behavior to broader client goals. Each of these domains is rated on a 7-point Likert scale (1 = very poor, 7 = excellent), culminating in a CMCS summary score. Prior research documents these domains’ strong scoring reliability, internal consistency, durability, concurrent validity, and predictive validity for client behavior (40). Competency in the domains was developed via observational and experiential learning activities (i.e., trainer demonstration, dyadic role-plays, and performance-based feedback). The temporal structure and learning strategies inherent in this coaching-to-criterion process were informed by documented preferences of a national sample of SUD program directors and staff (41), including scheduling of the sessions 7 days apart to allow for reflective inquiry and practice.

To conclude the coaching-to-criterion process, each staff member scheduled a 30-min individual meeting with CAAHS personnel to complete a structured role-play. Informed by established standardized patient methodology (42), role-plays involved CAAHS staff portraying a representative client character eligible to enroll in CM programming. Set in the circumstance of an intake visit following treatment court referral for services wherein a client earns a priming reinforcer, role-plays provided an opportunity for each staff member to demonstrate skill in the six CMCS fidelity domains. The lead author observed each role-play, rated CMCS domains in real-time, and provided immediate performance-based feedback. As applied in prior trials involving CM delivery by staff of community treatment programs (29, 43), implementation readiness was demonstrated by a configuration of CMCS ratings at or above an *a priori* benchmark (a rating of 4 on each of the six CMCS domains).

#### 3.1.3 Implementation phase

Upon demonstration of staff- and systems-level readiness, a 6-month period of pilot implementation commenced with continuous enrollment of eligible Emergence clients in the CM programming. As a primary source of implementation support, the lead author maintained biweekly consultative meetings with the facility’s two clinical supervisors to discuss the implementation experiences of their staff supervisees (with whom the supervisors maintained their supervision-as-usual practices) and troubleshoot any emerging challenges. Additionally, a tailored ‘CM resource library’ of implementation support materials was provided as communal access to training materials—including links to the online training modules, copies of coaching session slides and activity descriptions/instructions, implementation checklists, staff memory aids, and relevant publications. Emergence leaders were urged to incorporate the materials in regular staff meetings, as well as in the onboarding of future staff. To promote a sense of ownership of these materials, Emergence leaders and staff were encouraged to supplement the resource library with materials of their choosing, like demonstration videos depicting delivery of CM programming by familiar staff members.

#### 3.1.4 Sustainment phase

The concluding month in this year-long TA process consisted of a series of meetings among OHA, CAAHS, and Emergence representatives to discuss prospects for long-term sustainment of the CM programming. These discussions incorporated review of staff implementation experiences, clinic records of local effectiveness relative to historical control clients, implementation costs, and other considerations (e.g., other local treatment initiatives, prospective state legislative policies). This collective information was synthesized by CAAHS personnel into a summary report distributed to OHA and Emergence leaders. At a final meeting, CAAHS personnel sought a decision from Emergence leadership on whether its CM programming would, thereafter, be sustained, adapted, or discontinued.

### 3.2 CM programming at emergence

Via the aforementioned collaborative design process, customized CM programming to be implemented at Emergence was developed to be responsive to the stated organizational goal of increasing service engagement among treatment court referrals at its downtown Eugene facility.

As earlier mentioned, the CAAHS representative outlined options and offered recommendations while the core CM programming parameters to be implemented were ultimately determined by Emergence leadership, in accord with the organization’s available resources. Those CM programming parameters included: (1) enrollment of an eligible population of all persons referred for the facility’s SUD services by one of the local treatment courts; (2) one-time reinforcement of intake completion via a $50 voucher serving as a priming reinforcer, followed by iterative reinforcement for attendance at group-based Cognitive-Behavioral Therapy (CBT) sessions via $10 vouchers with additional weekly opportunity for a $10 “bonus” voucher tied to cumulative attendance for the week; (3) gift cards from a set of local vendors as the reinforcers, distributed to attendees at the conclusion of the corresponding SUD services (whether the completed intake assessment or attended group therapy session); and (4) a non-escalating, voucher-based reinforcement paradigm, occurring for a 12-week duration, with a per-client maximal earning of $530. As was expected, organization choices for CM programming parameters were influenced by local needs. One salient example was the choice of a non-escalating reinforcement paradigm, a more common choice by community-based organizations when reinforcing service engagement rather than substance abstinence [for which escalating reinforcement paradigms were originally developed (44, 45)]. In this case, this choice of CM paradigm reflected an organizational value at Emergence and among its leaders about the expected therapeutic benefits of its group-based CBT sessions, for which intrinsic client motivation is believed to increase over time. Another example of a CM programming parameter influenced by local circumstance was the choice to include a weekly opportunity for a “bonus” voucher, which was informed by well-known judicial views at the referring treatment courts that valued reliable participation in group therapy by justice-involved clients. In terms of day-to-day implementation procedures, Emergence staff monitored group CBT attendance via communal records, discussed with clients their prospective and earned reinforcers in weekly contacts, and ensured timely receipt of all earned reinforcers.

### 3.3 Measurement and outcomes

#### 3.3.1 Emergence staff and clientele

The demography and professional background of Emergence staff were not formally assessed, though this staff sample may otherwise be characterized as having been gainfully employed at Emergence in the set of aforementioned clinical or social service roles at the time of this implementation effort. These Emergence staff members identified and enrolled all eligible clients during the 6-month period of pilot implementation. To maintain client anonymity, CAAHS personnel were provided with available summary demographic data (i.e., age, gender, and ethnicity) for CM-enrolled clients during the pilot implementation period and clients similarly court-referred for SUD services during the previous year. Among this collective group of SUD service recipients, age ranged from 18 to 67 years with a mean of 38.07 (SD = 10.14). Client gender was distributed as 78% men, 21% women, and 1% transgender. Ethnic distribution of clients was 84% Caucasian, 8% Native American, 4% Black or African-American, 1% Pacific Islander, 1% Asian, and 3% Other. As for client ethnicity, 8% identified as Hispanic/Latino.

#### 3.3.2 Implementation outcomes

**Figure 1:**
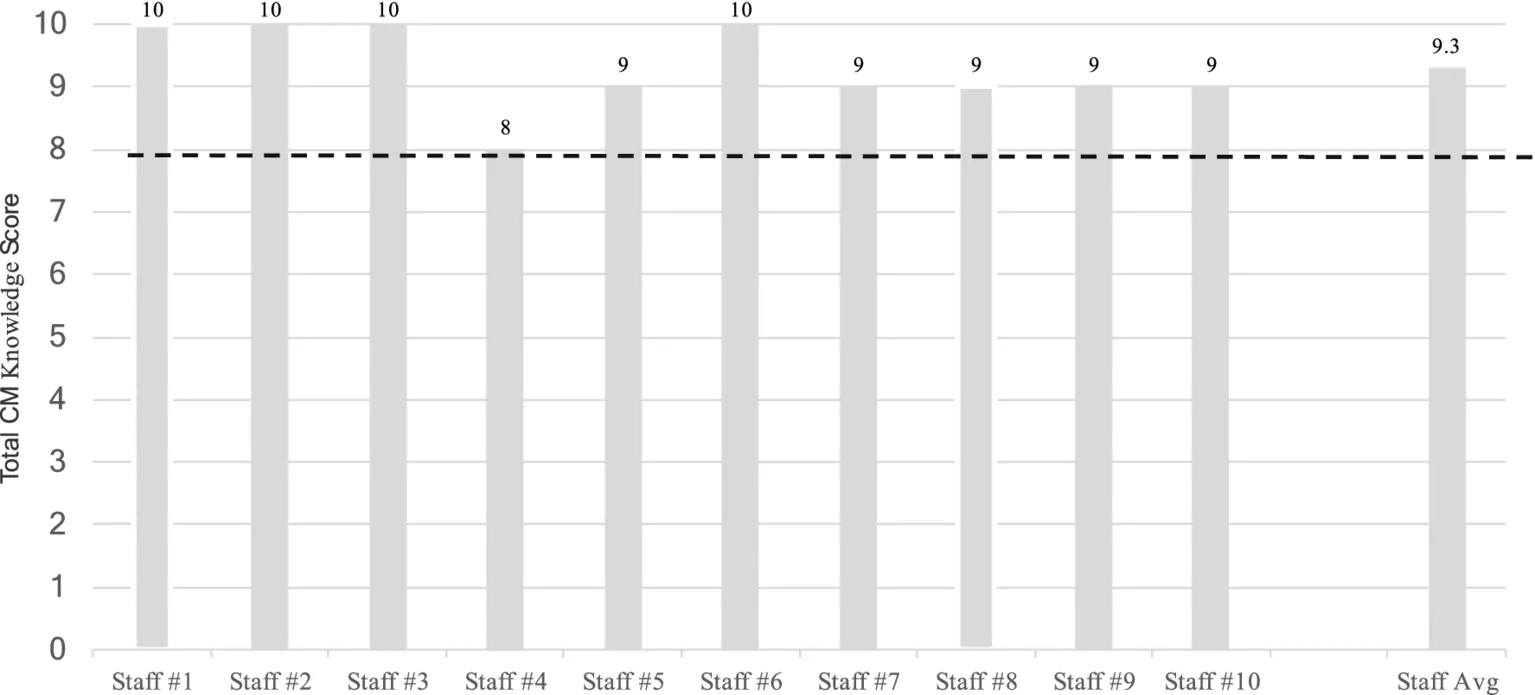
Knowledge-based preparation of Emergence staff to implement CM programming. CM knowledge of individual staff members assessed by a 10-item, multiple-choice test of common CM principles and practices completed by clinical supervisors, direct-care staff, and administrative support staff at conclusion of a role-specific online training module; total CM knowledge score reflects the number of correct responses to test questions (possible range 0–10), with the hatched line representing an *a priori* knowledge benchmark representing conceptual readiness to implement; staff avg. reflects the mean score (SD = 0.68) among the 10 Emergence staff members.

The CM knowledge and skillfulness of 10 Emergence staff were the two staff-level implementation outcomes assessed during the Preparation Phase. As demonstrated by individual Emergence staff members upon their completion of online training modules, the distribution of CM knowledge scores (*M* = 9.30, SD = 0.68) illustrated in Figure 1 suggests that, on average and across staff members, strong conceptual understanding of CM principles and practices was attained. This served as an index of setting readiness in advance of CM implementation and as a precursor for Emergence staff to develop skills to deliver their facility’s CM programming.

**Figure 2:**
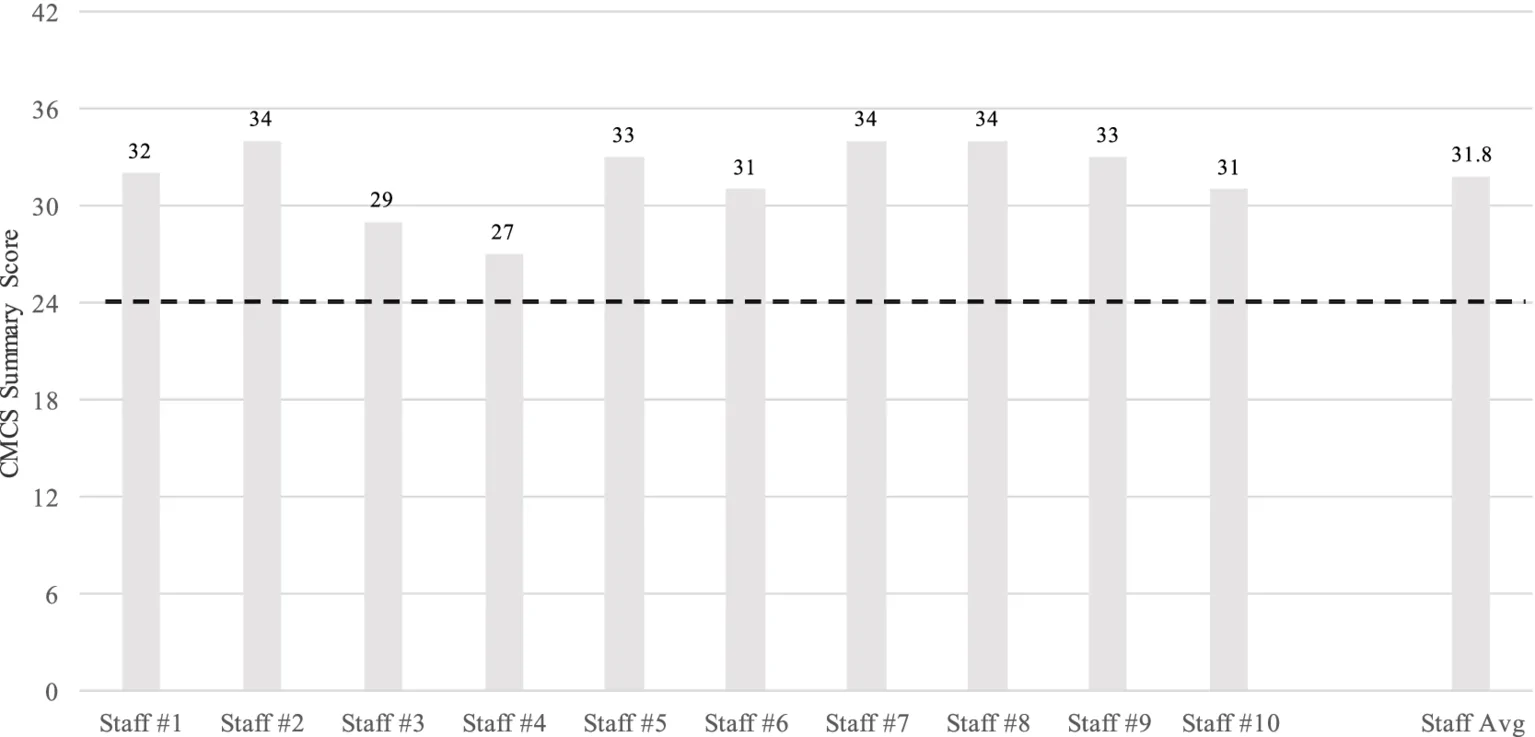
Skill-based preparation of Emergence staff to implement CM programming. CM skillfulness of individual staff members assessed during an observed, trainer-rated role-play with a standardized patient at conclusion of the coaching-to-criterion process; *CMCS summary score* reflects aggregation of ratings for six fidelity domains of the Contingency Management Competence Scale, each rated on a Likert scale (1 = very poor, 7 = excellent); Hatched line is the *a priori* fidelity benchmark representing readiness to implement, as employed in prior CM trials (29, 43); Staff avg. reflects the mean score (SD = 2.35) among the 10 Emergence staff members.

As demonstrated by individual Emergence staff members in observed role-play at the conclusion of their coaching-to-criterion process, all CMCS summary scores (ranging from 27 to 34) surpassed the *a priori* fidelity benchmark (see Figure 2). Moreover, the mean CMCS summary score of 31.80 (SD = 2.35) exceeded the noted benchmark by 3 + standard deviations. Taken together, this suggests that, on average and across staff, strong capability to skillfully deliver the customized CM programming was achieved. Notably, this distribution of CMCS summary scores is highly consistent with those observed in reports of our prior implementation efforts (29, 31), wherein post-training skillfulness was similarly assessed and subsequently sustained via supervision-as-usual efforts. It also offered a salient index of setting readiness in advance of the implementation of CM programming by Emergence staff.

#### 3.3.3 Clinical outcomes

Anonymized summary data were provided for all client referrals from the local treatment courts (*N* = 265), differentiated by timing of referral during the pilot CM implementation period (*n* = 53) or a historical control period (*n* = 212). Summary data included as group- and individual-level indices the following SUD service outcomes: (1) completion of a scheduled intake assessment, and (2) attendance at group CBT sessions occurring thrice-weekly. Group-level indices reflect the proportion across all clients of total scheduled appointments that were attended. Individual-level indices reflect the mean proportion of attended appointments per client, by weighting each client equally irrespective of the number of scheduled appointments. A pair of linear probability models, each with heteroscedasticity-robust standard errors, examined the effect of CM on the respective proportions of completed intake assessments and attended group CBT sessions. Table 2 lists summary statistics for each linear probability model.

**Table 2:** Clinical utility of CM programming.

	Historical control	CM-enrolled	Linear model summary statistics	
	Clients (*n* = 212)	Clients (*n* = 53)	Regression parameter	95% C.I.
*Outcome*				
Attended intake assessments				
Group-level	90/168 (53.6%)	100/152 (65.8%)		
Individual-level	*M* = 0.33 (SD = 0.45)	*M* = 0.88 (SD = 0.26)	*b* = 0.54***, SE = 0.05, *z* = 11.56	[0.45, 0.64]
Attended therapy groups				
Group-level	2,798/4,094 (68.3%)	3,577/4,743 (75.4%)		
Individual-level	*M* = 0.38 (SD = 0.43)	*M* = 0.49 (SD = 0.40)	*b* = 0.11^t^, SE = 0.06, *z* = 1.80	[0.01, 0.23]
Fulfilled SUD service requirements				
Group-level	22/83 (26.3%)	21/57 (36.8%)		

Linear probability models, each with heteroscedasticity-robust standard errors and inclusive of all clients (*N* = 265), examined the effects of CM treatment on the proportions of completed intake assessments and attended group therapy sessions. Historical Control clients were referred for SUD services the year before CM programming availability, whereas CM-Enrolled clients were referred during a 6-month pilot implementation period; Group-level summary statistics reflect data aggregated across all individuals referred by a local treatment court for SUD services during a 6-month period; Individual-level statistics reflect the average proportion of attended visits per individual; Data concerning fulfillment of court-assigned SUD service requirements were collected at group-level only; ***p < 0.001, ^t^trend toward statistical significance (*p* = 0.072).

For completed intake appointments, group-level analysis revealed a salient influence of CM intervention—with CM-enrolled clients completing a greater overall proportion of intake assessments (66%) than historical controls (54%). As for individual-level indices, the linear probability model revealed a statistically significant effect of CM intervention (*p* < 0.001, see Table 2). In descriptive terms, this was a robust effect with the intake completion rate among CM-enrolled clients (88%) more than doubling that of historical controls (33%). Unsurprisingly, this translated to a very large effect size (*d* = 1.31). For group CBT attendance, group-level analysis revealed a lesser influence of CM intervention, though CM-enrolled clients still attended a greater overall proportion of group sessions (75%) than historical controls (68%). As for individual-level indices, the linear probability model did not detect a statistically significant effect of CM intervention although there was a trend toward statistical significance (*p* = 0.072, see Table 2), with a group CBT attendance rate among enrolled clients (49%) exceeding that of historical controls (38%). This translated to a small effect size (*d* = 0.26).

Informed by clinical observations at Emergence, available clinic data at the conclusion of the 6-month pilot implementation period enabled group-level analysis of the rate at which clients had fulfilled court-assigned SUD service requirements. Consistent with the voiced observations of Emergence leaders and staff, this group-level analysis revealed a secondary (untargeted) influence of CM intervention wherein, after 6 months, a greater proportion of CM-enrolled clients had fulfilled court-assigned SUD service requirements (37%) relative to historical controls (26%). An anecdotal observation shared by Emergence staff with facility leaders was a palpable improvement in staff-client rapport that permeated the milieu and collective services.

#### 3.3.4 Sustainment decision

At the conclusion of pilot implementation, a summary report was generated and distributed to Emergence leadership and partners at OHA. The report contained a process description of the aforementioned TA process and results of descriptive analyses applied to available implementation and clinical outcomes. Upon its review, representatives from Emergence, OHA, and CAAHS met to discuss a disposition for the CM programming. This meeting discussion focused on feasibility issues of the CM programming, including its fiscal and logistical compatibility at the Emergence facility, as well as its clinical utility. A consensus-based decision was reached among Emergence leaders to sustain the CM programming without modification to its core parameters (i.e., client group, target behavior, earned reinforcers, and reinforcement paradigm). Based on the report of Emergence leaders, the CM programming remains in place as of this writing. Table 3 offers a retrospective account of this sustainment decision.

**Table 3:** Retrospective account of the SUD organization’s sustainment decision.

“From the organizational perspective at Emergence, the decision to sustain CM was more straightforward than might be assumed. Rather than relying only on end-of-pilot analysis, the decision emerged through ongoing observation of program performance during implementation. Clear improvements in attendance, engagement, and overall program tone became evident early in the pilot period. These observed changes provided sufficient justification to continue the model. Absent those improvements, sustainment would not have been pursued Among the factors considered, increased client engagement was the most important. Group attendance improved in a way that staff could observe directly. Clients who might otherwise have disengaged were showing up more consistently, and many connected their attendance to the incentives. A particularly notable change occurred during intake. Offering a $50 incentive for completing all steps, including ASAM assessment, orientation, and initial urinalysis, led to near-universal completion. Previously, many clients left before finishing this process Operational and financial considerations were also part of the decision, but became less of a concern over time. Initial funding for incentives was limited, yet increased attendance and participation supported the program in a way that offset those costs. Systems for tracking and distributing incentives were developed during implementation and functioned reliably, reinforcing confidence that the model could be maintained within routine operations Emergence also observed meaningful secondary effects in the clinical environment. In routine practice, treatment adjustments for non-adherence often involve increasing structure or accountability. CM introduced a consistent way to reinforce positive behavior, which was well received by both staff and clients. Staff reported stronger rapport with clients and a more positive atmosphere within the treatment setting At the same time, CM fit well within the organization’s existing approach to care. The model is structured when delivered with fidelity and aligned with other structured interventions already in place. It complemented cognitive behavioral programming and was consistent with the expectations of treatment court partners, who were supportive of incorporating more reinforcement-based strategies Taken together, these factors led to a clear and unanimous decision to sustain CM. Since the pilot period, CM has been integrated into routine operations and is introduced at intake alongside other core services. Little drift has been observed, and fidelity has been maintained through standard supervision practices. The organization also expanded CM to an additional program serving justice-involved clients, reflecting confidence in its applicability across settings Overall, Emergence’s experience suggests that CM can be implemented and sustained in real-world treatment settings at manageable cost and with meaningful clinical benefit.”

This retrospective account contains the full, unedited remarks of the SUD organization’s executive director, offered during the drafting of this manuscript—2 years after the original sustainment decision.

## 4 Discussion

Governed by the EPIS framework (33), this community case study details efforts of a continuing interagency partnership in Oregon wherein a university-based intermediary purveyor organization replicated prior successes applying this comprehensive, CM-focused TA approach. This involved the collaborative design of, preparation of staff and systems for, and sustainable implementation of customized CM programming by an organization targeting improved service engagement at its SUD facility among justice-involved clientele. Herein, we have documented: (1) data-based preparation of staff, including attainment of established benchmarks for CM knowledge and skillfulness, in advance of delivering the CM programming; (2) clinical utility of the CM programming among CM-enrolled clients, with a completion rate for intake assessments that more than doubled as well as an 11% increase in subsequent group CBT attendance; (3) a secondary therapeutic benefit reflected by an aggregated 11% increase in fulfillment of court-assigned SUD service requirements among CM-enrolled clientele; and (4) an eventual decision by organization leaders to sustain the CM programming as part of routine facility services. Notably, each of these findings broadly replicate those of our prior successes with offering this TA approach to aid community SUD organizations in achieving sustainable implementation of clinically useful CM programming (28, 29, 31, 46).

Given continuing efforts to identify viable funding mechanisms for CM implementation, a salient aspect of this community case study is how the interagency partnership arranged to fund the described TA process and implementation expenses. As the primary sponsor, OHA utilized SOR funds to contract with the CAAHS team for TA provision. Regarding direct implementation costs (i.e., gift cards as tangible reinforcers and staff time), Emergence bore the full responsibility of these expenses. Accordingly, per-client maximal earnings in the CM programming were a salient metric tracked throughout its collaborative design. At $530, this metric fell within the $750 per-patient annual limit outlined in a SAMHSA Advisory report (27), which may interest those who would consider replication with greater reliance on federal funding sources.

Cost considerations in the design of this CM programming also help explain the observed pattern of clinical impacts. A $50 priming reinforcer applied to intake completion was associated with a pronounced effect size (*d* = 1.31), whereas $10 reinforcers applied longitudinally to group CBT attendance (for each of the thrice-weekly groups and a potential weekly cumulative “bonus”) were associated with a lesser effect size (*d* = 0.26). This pattern of clinical effects is intuitive, and also broadly consistent with findings from a review of voucher-based reinforcement that note reinforcer magnitude as a moderator of clinical effectiveness (16). To that end, it is conceivable that increasing the magnitude of the reinforcers for group attendance may well have enhanced the corresponding effect size. Similarly, it is possible that application of an escalating voucher paradigm may have altered the effect size associated with group attendance, though it should be noted that the origins and continued recommendation of this paradigm (47) are tied specifically to CM protocols that target substance abstinence. In any event, these options were among those presented to Emergence leaders during the collaborative design process and were discarded due to incongruence with the organizational goals, values, and resources. As is evident in the executive director’s retrospective account of the CM sustainment decision, costs and magnitude of clinical effects are two of a myriad of competing considerations that organization leaders must weigh when considering sustainable implementation of CM. In this case, the planned resource allocation and resulting pattern of clinical effects were consistent with a stated priority voiced by Emergence leaders from project outset—to, first and foremost, improve initial SUD service linkage among treatment court referrals. Indeed, this voiced priority of Emergence leaders not only addressed a longstanding local organizational challenge but also aligns with national findings regarding the care cascade for justice-involved populations (48).

As has been observed in prior applications of this CM-focused TA approach (29, 31), secondary therapeutic benefits were observed in unincentivized client behaviors. Specifically,

an increase was observed in the rate at which CM-enrolled clients fulfilled their court-assigned SUD service requirements—an outcome with profound potential for state-level public health impact.

Notably, and as reported by the Oregon Criminal Justice Commission (49), over a multi-year period that corresponds with the timeline for this case study, justice-involved Oregonians who fulfilled court-assigned requirements demonstrated a much lower recidivism rate (25%) relative to those who did not fulfilling such requirements (64%). For those who work with justice-involved clientele, we hope this set of observations invites innovation about future applications of CM that may be implemented with hard-to-reach, often hard-to-treat populations. As for other apparent benefits to the milieu at this facility, anecdotal staff reports to their clinical supervisors suggest that staff were quick to perceive facility-wide improvement in staff-client rapport following the initial introduction of the CM programming, an observation that extant research has long recognized as an accelerant for workforce adoption of CM (50, 51). The benefits to an organization of such an impact are likely pervasive, and merit further attention in future research. Whether such unplanned yet beneficial observations are considered at a societal or individual level, they strengthen the rationale for organizations like Emergence to customize CM programming as a means of achieving clinically meaningful outcomes via sustainable implementation.

One aspect of this community case study for which journal readership may particularly benefit is a greater understanding of how SUD program leaders forge sustainment decisions. Some academic researchers may presume a singular basis for such decisions, driven by an allegiance to the metrics of clinical impact reported in controlled trials. Such trials typically include unrealistic sources of control that do not exist outside of laboratories or research clinics, leaving the very real task of arriving at a sustainment decision as one that requires a pragmatic balancing of fiscal, organizational, and clinical considerations. Interestingly, the account of this sustainment decision brings to mind the seminal work of Everett Rogers (52), and his oft-cited attributes known to spur greater adoption of innovations: relative advantage, compatibility, complexity (notably, an inverse predictor of adoption), trialability, and observability. Each of these attributes is identifiable within the conceptual weaving of this executive director’s retrospective account of a decision to sustain CM programming. Future CM implementation efforts may consider offering similar emphasis on such organizational decision-making processes.

## 5 Acknowledgements and conclusion

As a community case study, the current study does include clear methodological caveats. Prominent among these are the single-site design, which may limit generalizability of the findings, and the quasi-experimental design employed to evaluate clinical impacts that does not control for the potential influence of third variables or a Hawthorne effect. Regarding both of these caveats, it is worth noting that the aforementioned TA approach has previously been applied with several other SUD organizations, in Oregon and elsewhere, with comparable success. An additional caveat stems from reliance on the SUD organization to provide anonymized client records to enable an independent evaluation of the CM programming. Limits of real-world clinical record-keeping, along with the organization’s concerted effort to maintain anonymity of their clients, constrained the scope and sophistication with which clinical impacts of this CM programming were evaluated. Moreover, this community case study did not include direct assessment of CM programming delivery by Emergence staff in their day-to-day client encounters. As detailed, the data-based preparatory process for these staff was extensive—encompassing initial didactic training and subsequent skills-based coaching—and the resulting staff-based implementation outcomes suggest that Emergence staff members were well-prepared for interactions they would have with their clients. Furthermore, extant literature establishes that durability of benchmarks for CM knowledge and skillfulness, once attained, particularly when such initial preparation is followed by participation in an active supervision process (29, 43), as occurred at this SUD organization. This, and the additional consultative support and CM resource library provided by CAAHS, may only have augmented the quality with which Emergence staff delivered the CM programming, as was also suggested by qualitative observations of palpable changes to the milieu.

Caveats notwithstanding, this community case study offers a model whereby others may sustainably implement CM programming that is customized to local clinical needs and resources. In our continuing delivery of this CM-focused TA approach to date, two of its components—the initial use of a collaborative design process and subsequent staff preparation for CM delivery via a combination of self-paced, role-specific didactic modules and skills-focused participation in a coaching-to-criterion process—stand out as key ingredients. The collaborative design process pools theoretical and pragmatic expertise to design CM programming that is responsive to the clinical pain points of an organization, and, in so doing, seems to strengthen organizational commitment to implementation, evaluation, and eventual sustainment. Staff participation in a coaching-to-criterion process affords an organization the assurance that its individual and collective staff are conceptually knowledgeable about CM principles and practices, and ready to skillfully deliver a new, goal-oriented framework for working alongside and reinforcing clients. Aside from intuitive appeal, this framework for CM has been recurrently associated with secondary therapeutic benefits to the milieu and clinical gains in unincentivized areas of client functioning. Rather than a burden of “one more thing” a clinician must do, CM may instead become realized as a tool that clinicians can utilize to foster and generalize therapeutic growth among their clientele. We hope this community case study serves as a useful model for others seeking to incorporate CM into their settings.

## Data Availability

The raw data supporting the conclusions of this article will be made available by the authors, without undue reservation.

## References

[1] McCarty DJ Chandler RK. Understanding the importance of organizational and system variables on addiction treatment services within criminal justice settings. Drug Alcohol Depend. (2009) 103:S91–3. doi: 10.1016/j.drugalcdep.2009.03.001, 19356862

[2] Beaudry G Yu R Perry AE Fazel S. Effectiveness of psychological interventions in prison to reduce recidivism: a systematice review and meta-analysis of randomized controlled trials. Lancet Psychiatry. (2021) 8:759–73. doi: 10.1016/S2215-0366(21)00170-X, 34419185 PMC8376657

[3] Office of National Drug Control Policy. National Drug Control Budget: FY 2025 Funding Highlights. Washington, D.C.: The White House, Executive Office of the President (2024).

[4] Office of National Drug Control Policy. National Drug Control Budget: Budget Highlights Fiscal Year 2026.The White House, Office of the President (2025).

[5] Proctor E Hooley C Morse A McCrary S Kim H Kohl PL. Intermediary/purveyor organizations for evidence-based interventions in the U.S. child mental health: characteristics and implementation strategies. Implement Sci. (2019) 14:3. doi: 10.1186/s13012-018-0845-3, 30642342 PMC6332855

[6] Prendergast M Hall EA Roll JM Warda U. Use of vouchers to reinforce abstinence and positive behaviors among clients in a drug court treatment program. J Subst Abus Treat. (2008) 35:125–36. doi: 10.1016/j.jsat.2007.09.001, 17997267 PMC2527867

[7] Friedman PD Taxman FS Henderson CE. Evidence-based treatment practices for drug-involved adults in the criminal justice system. J Subst Abus Treat. (2007) 32:267–77. doi: 10.1016/j.jsat.2006.12.020, 17383551 PMC1885209

[8] Little GL Robinson KD. Moral Reconation therapy: a systematic step-by-step treatment system for treatment of resistant clients. Psychol Rep. (1988) 62:135–51. doi: 10.2466/pr0.1988.62.1.135, 3283816

[9] Higgins ST Silverman K Heil SH. Contingency Management in Substance Abuse Treatment. New York: Guilford (2008).

[10] Rudes DS Portillo S Murphy A Rhodes A Stitzer ML Loungo P . Adding positive reinforcements in a criminal justice setting: acceptability and feasibility. J Subst Abus Treat. (2011) 42:260–70. doi: 10.1016/j.jsat.2011.08.002 PMC3674847 21940135

[11] DeFulio A Stitzer ML Roll JM Petry NM Nuzzo PA Schwartz RP . Criminal justice referral and incentives in outpatient substance abuse treatment. J Subst Abus Treat. (2013) 45:70–5. doi: 10.1016/j.jsat.2012.12.012, 23433822 PMC3645315

[12] Petry NM Rash CJ Easton CJ. Contingency management treatment in substance abusers with and without legal problems. J Am Acad Psychiatry Law. (2011) 39:370–8. 21908754 PMC3616315

[13] Sloas L Murphy A Wooditch A Taxman FS. Assessing the use and impact of points and rewards across four federal probation districts: a contingency management approach. Vict Offenders. (2019) 14:811–31. doi: 10.1080/15564886.2019.1656691, 33041726 PMC7545962

[14] Brown HD DeFulio A. Contingency management for the treatment of methamphetamine disorder: a systematic review. Drug Alcohol Depend. (2020) 216:108307. doi: 10.1016/j.drugalcdep.2020.108307 33007699

[15] Benishek LA Dugosh KL Kirby KC Matejkowski J Clements NT Seymour BL . Prize-based contingency management for the treatment of substance abusers: a meta-analysis. Addiction. (2014) 109:1426–36. doi: 10.1111/add.12589, 24750232 PMC4203362

[16] Lussier JP Heil SH Mongeon JA Badger GJ Higgins ST. A meta-analysis of voucher-based reinforcement therapy for substance use disorders. Addiction. (2006) 101:192–203. doi: 10.1111/j.1360-0443.2006.01311.x, 16445548

[17] Griffith JD Rowan-Szal GA Roark RR Simpson DD. Contingency management in outpatient methadone treatment: a meta-analysis. Drug Alcohol Depend. (2000) 58:55–66. doi: 10.1016/S0376-8716(99)00068-X, 10669055

[18] De Crescenzo F Ciabattini M D'Alo GL De Giorgi R Del Giovane C Janiri L . Comparative efficacy and acceptability of psychosocial interventions for individuals with cocaine and amphetamine addiction: a systematic review and network meta-analysis. PLoS Med. (2018) 15:e1002715. doi: 10.1371/journal.pmed.1002715 30586362 PMC6306153

[19] Petry NM Alessi SM Olmstead TA Rash CJ Zajac K. Contingency management treatment for substance use disorders: how far has it come, and where does it need to go? Psychol Addict Behav. (2017) 31:897–906. doi: 10.1037/adb0000287, 28639812 PMC5714694

[20] Marlowe DB Wong CJ. "Contingency management in adult criminal drug courts". In: Higgins ST Silverman K Heil SH, editors. Contingency Management in Substance abuse Treatment. New York: Guilford Press (2008). p. 334–54.

[21] White WL Kelly JF. Toward a solution-focused addiction science. Front Public Health. (2025) 13:1701524. doi: 10.3389/fpubh.2025.1701524, 41377714 PMC12685922

[22] Hartzler B Lash SJ Roll JM. Contingency management in substance abuse treatment: a structured review of the evidence for its transportability. Drug Alcohol Depend. (2012) 122:1–10. doi: 10.1016/j.drugalcdep.2011.11.011, 22153943 PMC3307900

[23] Rawson RA Erath TG Chalk M Clark HW McDaid C Wattenberg SA . Contingency management for stimulant use disorder: progress, challenges, and recommendations. J Ambul Care Manage. (2023) 46:152–9. doi: 10.1097/JAC.0000000000000450 36745163

[24] Petry NM DePhillipis D Rash CJ Drapkin M McKay JR. Nationwide dissemination of contingency management: the veterans administration initiative. Am J Addict. (2014) 23:205–10. doi: 10.1111/j.1521-0391.2014.12092.x, 24724876 PMC3986725

[25] Becker SJ Murphy CM Hartzler B Rash CJ Janssen T Roosa M . Project MIMIC (maximizing implementation of motivational incentives in clinics): a cluster-randomized type 3 hybrid effectiveness-implementation trial. Addict Sci Clin Pract. (2021) 16:61. doi: 10.1186/s13722-021-00268-0, 34635178 PMC8505014

[26] Cooke A Stack E Peng A Cook R Hartzler B Leichtling G . The peers expanding engagement in stimulant harm reduction with contingency management study: a protocol paper. Addict Sci Clin Pract. (2025) 20:48. doi: 10.1186/s13722-025-00577-8, 40495203 PMC12150483

[27] SAMHSA. Using SAMHSA Funds To Implement Evidence-Based Contingency Management Services. Rockville: Substance Abuse and Mental Health Services Administration (2025).

[28] Hartzler B. Building a bonfire that remains stoked: sustainment of a contingency management intervention through collaborative design. Subst Abuse Treat Prev Policy. (2015) 10:30. doi: 10.1186/s13011-015-0027-0 26243132 PMC4526292

[29] Hartzler B Jackson TR Jones BE Beadnell B Calsyn DA. Disseminating contingency management: impacts of staff training and implementation at an opiate treatment program. J Subst Abus Treat. (2014) 46:429–38. doi: 10.1016/j.jsat.2013.12.007, 24462242 PMC3966904

[30] Hartzler B Beadnell B Donovan DM. Predictive validity of addiction treatment clinicians’ post-training contingency management skills for subsequent clinical outcomes. J Subst Abus Treat. (2017) 72:126–33. doi: 10.1016/j.jsat.2015.11.010, 26733276 PMC4882285

[31] Hartzler B Gray K Marx M Kirk-Lewis K Payne-Smith K McIlveen JW. Implementing contingency management to address stimulant use. J. Subst. Use Addict. Treat. (2023):151. doi: 10.1016/j.josat.2022.208941

[32] Cross-Technology Transfer Center Workgroup on Virtual Learning. Virtual reality for behavioral health workforce development in the era of COVID-19. J Subst Abus Treat. (2021) 121:108157. doi: 10.1016/j.jsat.2020.108157, 33223379 PMC7545203

[33] Aarons GA Hurlburt M McCue HS. Advancing a conceptual model of evidence-based practice implementation in public service sectors. Adm Policy Ment Health Ment Health Serv. (2011) 38:4–23. doi: 10.1007/s10488-010-0327-7, 21197565 PMC3025110

[34] Lyon AR Koerner K. User-centered design for psychosocial intervention development and implementation. Clin Psychol. (2016) 23:180–200. doi: 10.1111/cpsp.12154, 29456295 PMC5812700

[35] Chambers DA Glasgow RE Stange KC. The dynamic sustainability framework: addressing the paradox of sustainment and ongoing change. Implement Sci. (2013) 8:117 doi: 10.1186/1748-5908-8-117 24088228 PMC3852739

[36] Kellogg SH Burns M Coleman P Stitzer ML Wale JB Kreek MJ. Something of value: the introduction of contingency management interventions into the new York City health and hospital addiction treatment service. J Subst Abus Treat. (2005) 28:57–65. doi: 10.1016/j.jsat.2004.10.007, 15723733

[37] Hartzler B Peng L Cooke A Kunkel L Stack E Leahy J . Reason to expand the contingency management workforce: coaching-to-criterion results for addiction professionals and peers. Subst Use Addict. (2025) 4:29767342251363007. doi: 10.1177/29767342251363007 PMC13137054 40908764

[38] Petry NM Alessi SM Ledgerwood DM Sierra S. Psychometric properties of the contingency management competence scale. Drug Alcohol Depend. (2010) 109:167–74. doi: 10.1016/j.drugalcdep.2009.12.027, 20149950 PMC2875270

[39] Petry NM Ledgerwood DM. The Contingency Management Competence Scale for Reinforcing Attendance. Farmington: University of Connecticut Health Center (2010).

[40] Hartzler B. Adapting the helpful responses questionnaire to assess communication skills involved in delivering contingency management: preliminary psychometrics. J Subst Abus Treat. (2015) 55:52–7. doi: 10.1016/j.jsat.2015.02.006, 25770870 PMC4456271

[41] Hartzler B Rabun C. Training addiction professionals in empirically-supported treatments: perspectives from the treatment community. Subst Abus. (2014) 35:30–6. doi: 10.1080/08897077.2013.789816, 24588290 PMC3947611

[42] Fairburn CG Cooper Z. Therapist competence, therapy quality, and therapist training. Behav Res Ther. (2011) 49:373–8. doi: 10.1016/j.brat.2011.03.005, 21492829 PMC3112491

[43] Petry NM Alessi SM Ledgerwood D m. Contingency management delivered by community therapists in outpatient settings. Drug Alcohol Depend. (2012) 122:86–92. doi: 10.1016/j.drugalcdep.2011.09.015, 21981991 PMC3290694

[44] Higgins ST Budney AJ Bickel WK Hughes JR Roerg F Badger GJ. Achieving cocaine abstinence with a behavioral approach. Am J Psychiatry. (1993) 150:763–9. doi: 10.1176/ajp.150.5.763, 8480823

[45] Higgins ST Budney AJ Bickel WK Hughes J Foerg FE Badger GJ. Incentives improve outcome in outpatient behavioral treatment of cocaine dependence. Arch Gen Psychiatry. (1994) 51:568–76. doi: 10.1001/archpsyc.1994.03950070060011, 8031230

[46] Hartzler B Peavey KM Jackson TR Carney M. Finding harmony so the music plays on: pragmatic trial design considerations to promote organizational sustainment of an empirically-supported behavior therapy. Addict Sci Clin Pract. (2016) 11:2. doi: 10.1186/s13722-016-0049-6 26801244 PMC4724112

[47] Rash CJ Black S Parent SC Erath TG McDonell MG. Data-driven contingency management incentive magnitudes: a review. JAMA Psychiatry. (2025) 82:940–5. doi: 10.1001/jamapsychiatry.2025.1341, 40601332

[48] Clark KJ Viglione J Sneed R Ramezani N Taxman FS Johnson JE. Cascade of care for substance use and mental health disorders for justice-involved populations. J Subst Use Addict Treat. (2024) 167:209488. doi: 10.1016/j.josat.2024.209488, 39181506 PMC11527580

[49] Oregon Criminal Justice Commission. Recidivism in Oregon Specialty Courts. Salem: Oregon Criminal Justice Commission (2023).

[50] Aletraris L Shelton JS Roman PM. Counselor attitudes toward contingency management for substance use disorder: effectiveness, acceptability, and endorsement of incentives for treatment attendance and abstinence. J Subst Abus Treat. (2015) 50:41–8. doi: 10.1016/j.jsat.2015.04.012 PMC4561006 26001821

[51] Ducharme LJ Knudsen HK Abraham AJ Roman PM. Counselor attitudes toward the use of motivational incentives in addiction treatment. Am J Addict. (2010) 19:496–503. doi: 10.1111/j.1521-0391.2010.00081.x, 20958844 PMC2959187

[52] Rogers EM. Diffusion of Innovations. New York: The Free Press (2003).

